# Rickettsia Lipid A Biosynthesis Utilizes the Late Acyltransferase LpxJ for Secondary Fatty Acid Addition

**DOI:** 10.1128/JB.00334-18

**Published:** 2018-09-10

**Authors:** Mark L. Guillotte, Joseph J. Gillespie, Courtney E. Chandler, M. Sayeedur Rahman, Robert K. Ernst, Abdu F. Azad

**Affiliations:** a Department of Microbiology and Immunology, School of Medicine, University of Maryland, Baltimore, Maryland, USA; b Department of Microbial Pathogenesis, School of Dentistry, University of Maryland, Baltimore, Maryland, USA; Princeton University

**Keywords:** LPS, lipid A, LpxJ, pathogenesis, Rickettsia, outer membrane

## Abstract

Members of the Rickettsia genus are obligate intracellular, Gram-negative coccobacilli that infect mammalian and arthropod hosts. Several rickettsial species are human pathogens and are transmitted by blood-feeding arthropods. In Gram-negative parasites, the outer membrane (OM) sits at the nexus of the host-pathogen interaction and is rich in lipopolysaccharide (LPS). The lipid A component of LPS anchors the molecule to the bacterial surface and is an endotoxic agonist of Toll-like receptor 4 (TLR4). Despite the apparent importance of lipid A in maintaining OM integrity, as well as its inflammatory potential during infection, this molecule is poorly characterized in Rickettsia pathogens. In this work, we have identified and characterized new members of the recently discovered LpxJ family of lipid A acyltransferases in both Rickettsia typhi and Rickettsia rickettsii, the etiological agents of murine typhus and Rocky Mountain spotted fever, respectively. Our results demonstrate that these enzymes catalyze the addition of a secondary acyl chain (C_14_/C_16_) to the 3′-linked primary acyl chain of the lipid A moiety in the final steps of the Raetz pathway of lipid A biosynthesis. Since lipid A architecture is fundamental to bacterial OM integrity, we believe that rickettsial LpxJ may be important in maintaining membrane dynamics to facilitate molecular interactions at the host-pathogen interface that are required for adhesion and invasion of mammalian cells. This work contributes to our understanding of rickettsial outer membrane physiology and sets a foundation for further exploration of the envelope and its role in pathogenesis.

**IMPORTANCE** Lipopolysaccharide (LPS) triggers an inflammatory response through the TLR4-MD2 receptor complex and inflammatory caspases, a process mediated by the lipid A moiety of LPS. Species of Rickettsia directly engage both extracellular and intracellular immunosurveillance, yet little is known about rickettsial lipid A. Here, we demonstrate that the alternative lipid A acyltransferase, LpxJ, from Rickettsia typhi and R. rickettsii catalyzes the addition of C_16_ fatty acid chains into the lipid A 3′-linked primary acyl chain, accounting for major structural differences relative to the highly inflammatory lipid A of Escherichia coli.

## Abstract (precis)

Lipopolysaccharide (LPS) triggers an inflammatory response through the TLR4-MD2 receptor complex and inflammatory caspases, a process mediated by the lipid A moiety of LPS. Species of Rickettsia directly engage both extracellular and intracellular immunosurveillance, yet little is known about rickettsial lipid A. Here, we demonstrate that the alternative lipid A acyltransferase, LpxJ, from Rickettsia typhi and R. rickettsii catalyzes the addition of C_16_ fatty acid chains into the lipid A 3′-linked primary acyl chain, accounting for major structural differences relative to the highly inflammatory lipid A of Escherichia coli.

## INTRODUCTION

Species of Rickettsia (Rickettsiales: Alphaproteobacteria) are Gram-negative obligate intracellular parasites of a vast range of eukaryotes (1). While mechanisms for host cell invasion are variable across rickettsial lineages (2), all rickettsiae lyse the host phagocytic vacuole and reside primarily in the host cytosol (3). Dependent on a plethora of host metabolites, rickettsiae have a diminished metabolic capability relative to that of free-living and facultative intracellular bacteria, as well as vacuolar obligate intracellular species (4). Thus, while rickettsiae vary in their abilities to infect vertebrates and cause pathogenesis, all species are metabolic parasites of the eukaryotic cytoplasm.

Remarkably, despite the lack of glycolytic enzymes, rickettsiae synthesize a typical Gram-negative bacterial cell envelope (5–7). The inner membrane (IM) and outer membrane (OM) are separated by a relatively thin peptidoglycan layer (8), which contains diaminopimelate in the stem peptide (9). The OM is asymmetric, with the inner leaflet composed mainly of glycerophospholipids (10, 11) and the outer leaflet predominantly composed of lipopolysaccharide (LPS) (12–14). As the interface connecting host to microbe, the rickettsial OM constituents are critically important not only for host cell invasion but also for mediating intracellular survival. The composition of surface proteins has been identified for several species (15–18), and nearly two dozen effectors are recognized as components of the secretome (19); however, the nonproteinaceous surface components of Rickettsia, including LPS, are critically understudied.

**FIG 1:**
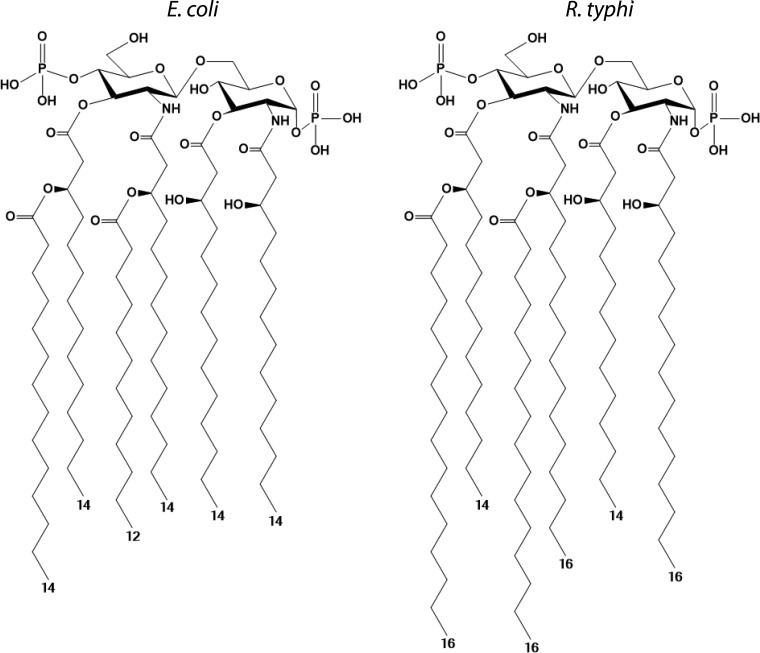
Lipid A structures of Escherichia coli and Rickettsia typhi.

LPS is highly antigenic in rickettsial infection (20, 21) and is composed of an outer O-antigen polysaccharide linked to a core oligosaccharide, which is anchored in the bacterial outer leaflet by lipid A (22). Lipid A is a pathogen-associated molecular pattern (PAMP) known for its ability to trigger an inflammatory response through its interaction with the Toll-like receptor 4/myeloid differentiation factor 2 (TLR4/MD2) complex, as well as activation of the noncanonical inflammasome through cytosolic caspases (Casp-4/Casp-5 in humans; Casp-11 in mice) (23–25). In a mouse model of rickettsiosis, TLR4/MD2 activation is critical for bacterial clearance (26, 27); however, little is known about the contribution of LPS to the inflammatory nature of Rickettsia infection (28, 29). Recent analysis of the lipid A from Rickettsia typhi revealed structural differences relative to the highly inflammatory lipid A of Escherichia coli though the potential of rickettsial lipid A to act as a TLR4 agonist remains unclear (30) (Fig. 1).

Our recent phylogenomics study indicated that rickettsiae contain a nearly conserved Raetz pathway for the synthesis of lipid A, lacking only the late acyltransferase LpxM (4). LpxM, which catalyzes the 3′ secondary acylation of 3-deoxy-d-*manno*-2-octulosonic acid (KDO_2_)-lauroyl-lipid IV_A_ (typically transferring myristate) subsequent to 2′ secondary acylation (carried out by LpxL), is absent in many bacteria, some of which alternatively carry a nonorthologous late acyltransferase named LpxJ (previously named DUF374) (31–33). LpxJ enzymes of Helicobacter pylori, Campylobacter jejuni, and Wolinella succinogenes (all Epsilonproteobacteria pathogens) catalyze 3′ secondary acylation but can or must do so prior to 2′ secondary acylation (LpxL) and even 3-deoxy-d-*manno*-octulosonic acid (KDO) transfer (carried out by the KDO transferase WaaA). Thus, LpxJ enzymes can be considered more functionally promiscuous than their LpxM counterparts. As LpxJ homologs are present in all Rickettsia genomes, with the R. typhi enzyme sharing 27% identity with H. pylori LpxJ, we reasoned that these enzymes complete the Raetz pathway for rickettsial lipid A biosynthesis and incorporate a C_16_ fatty acid chain as a 3′ secondary acylation (30).

Here, we provide enzymatic evidence that Rickettsia LpxJ complements E. coli LpxM mutants and carries out 3′ secondary acylation of lipid IV_A_ and lauroyl-lipid IV_A_. Additionally, targeted mutagenesis based on comparative analysis of >2,800 DUF374 family members with LpxJ homologs reveals residues critical for acylation. In line with prior work (31), our data demonstrate that divergent LpxJ and LpxM active sites both catalyze 3′ secondary acylation for lipid A biosynthesis and that LpxJ is a nonorthologous replacement of LpxM in a vast range of diverse bacteria. As lipid A architecture is fundamental to OM integrity in Gram-negative bacteria, our findings indicate that LpxJ may be important in maintaining ideal membrane dynamics to facilitate molecular interactions at the host-pathogen interface.

## RESULTS

### Rickettsia encodes a homolog of LpxJ.

Rickettsial comparative genomic analysis has identified a nearly complete Raetz pathway of lipid A biosynthesis (see Fig. S1 in the supplemental material). However, Rickettsia species do not encode any enzymes similar to LpxM (also known as MsbB). Since R. typhi (and probably all species of Rickettsia) produces hexa-acylated lipid A (30), we reasoned that a lipid A acyltransferase analogous to LpxM has escaped gene annotation within rickettsial genomes. In a report describing LpxJ, Rubin et al. identified a putative LpxJ homolog in R. rickettsii (27% similarity at the protein level) (31). We have further identified LpxJ family genes throughout the genus Rickettsia (Table 1) and have selected putative homologs from R. typhi (RT0047) (Fig. S2) and R. rickettsii (A1G_00705), here termed LpxJ^Rt^ and LpxJ^Rr^, respectively, for molecular characterization.

**TABLE 1:** Conservation between rickettsial LpxJ homologs

Rickettsia species (strain)	Locus tag	Homology^*a*^		E value
		% identity	% positive	
R. typhi (Wilmington)	RT0047	100	100	2E−161
R. prowazekii (Breinl)	H375_5410	98	99	8E−159
R. rickettsii (Sheila Smith)	A1G_00705	88	92	2E−140
R. felis (LSU)	JS55_00590	89	93	1E−142
R. akari (Hartford)	A1C_00645	85	91	1E−136
R. belli (RML Mogi)	RBEMOGI_1439	79	90	2E−129

a BLAST analysis was performed using R. typhi (Wilmington) LpxJ primary protein sequence as the query. Zero gaps were found in all query-subject alignments.

### LpxJ^Rt^ and LpxJ^Rr^ complement an E. coli LpxM mutant.

**FIG 2:**
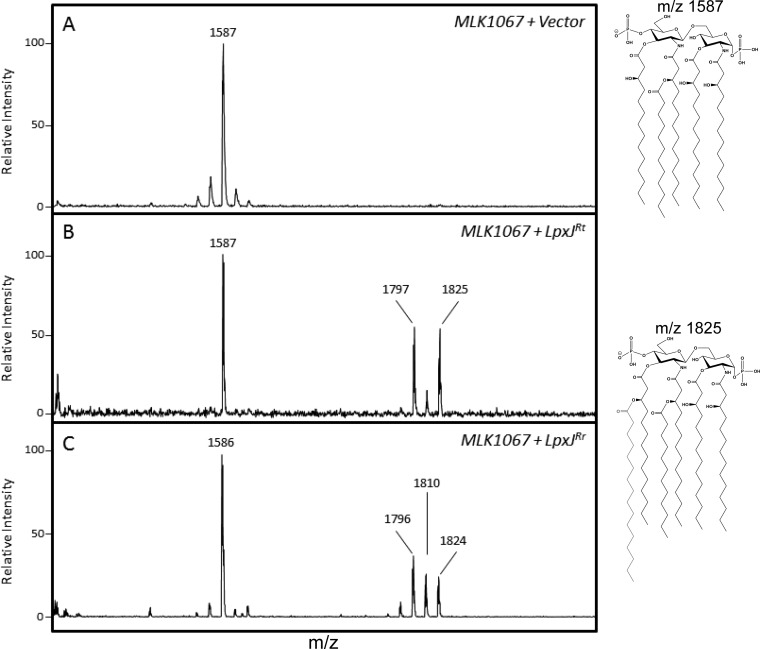
LpxJ^Rt^ and LpxJ^Rr^ complement the loss of LpxM in E. coli and restore a hexa-acylated lipid A phenotype. (A) E. coli mutant strain MLK1067 (Δ*lpxM*) produces mostly penta-acylated lipid A, corresponding to a major ion peak at *m/z* 1,587. (B and C) Expression of LpxJ^Rt^ (B) or LpxJ^Rr^ (C) restores a hexa-acylated lipid A phenotype by addition of C_14_ or C_16_ fatty acid corresponding to the molecular ion at *m/z* 1,797 or *m/z* 1,825, respectively.

**FIG 3:**
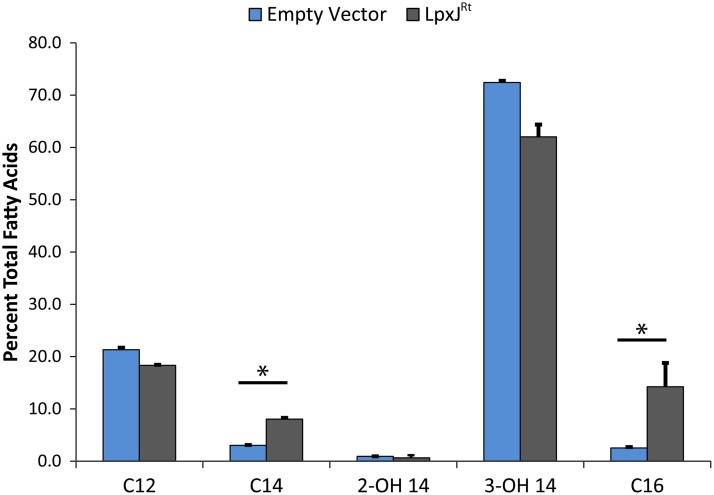
LpxJ transfers secondary C_14_ or C_16_ to the hydroxymyristate at the 3′ position. The fatty acid proportions present in the LPS isolated from E. coli strain MLK1067 carrying an empty vector or expressing *lpxJ* of R. typhi are given as percentages of the total identified fatty acids. A 4-fold increase of C_14_ and C_16_ addition was observed in the E. coli mutant complemented with *lpxJ^Rt^*. Analysis was run in biological triplicate from three isolated transformants and values were plotted ± standard deviations. *, *P* < 0.05, as determined by Student's *t* test.

In order to investigate the role of LpxJ in Rickettsia lipid A biosynthesis, we utilized a heterologous system in which acylation-deficient lipid A mutants of E. coli act as a reporter of enzyme function for exogenously expressed acyltransferases. We first expressed LpxJ^Rt^ and LpxJ^Rr^ in the *lpxM* mutant MLK1067 that elaborates predominately penta-acylated lipid A. After expression of rickettsial proteins was induced (Fig. S3), lipid A extractions were prepared and subjected to matrix-assisted laser desorption ionization–time of flight mass spectrometry (MALDI-TOF MS) analysis to determine if rickettsial LpxJ can complement the loss of *lpxM* and produce hexa-acyl lipid A. In comparison to results in untransformed MLK1067, we observed additional lipid A species of increased mass from cells expressing LpxJ^Rt^ and LpxJ^Rr^ but no change from cultures transformed with an empty plasmid vector (Fig. 2). The ions at *m/z* 1,797 and *m/z* 1,825 represent the addition of C_14_ (Δ*m/z* 210) or C_16_ (Δ*m/z* 238), respectively, to the parental penta-acylated lipid A (*m/z* 1,587). MALDI-TOF MS results for LpxJ^Rt^ were confirmed using gas chromatography (GC). Fatty acid peaks were identified by comparison to commercially available bacterial acid methyl ester (BAME) standards. The amount of each fatty acid present in lipid A was calculated by comparison to an internal pentadecanoic acid (C_15_) standard over three biological replicates. LpxJ^Rt^-overexpressing cells show a 4-fold increase in the total amount of C_14_ and C_16_ (Fig. 3). Taken together, these data indicate that LpxJ^Rt^ and LpxJ^Rr^ are bona fide acyltransferases in the lipid A pathway.

### LpxJ^Rt^ activity is independent of LpxL activity.

**FIG 4:**
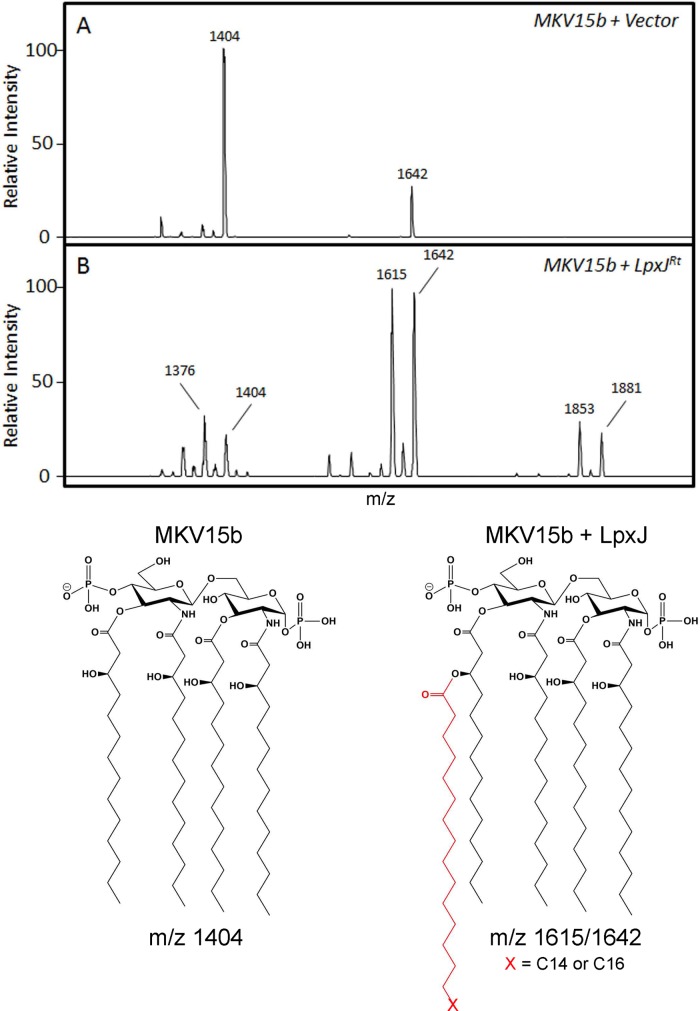
Acylation of lipid A by LpxJ^Rt^ does not depend upon prior secondary acylation. MALDI-TOF MS analysis was performed of lipid A from E. coli strain MKV15b (Δ*lpxM* Δ*lpxL* Δ*lpxP*) (35) transformed with empty vector (A) or expressing LpxJ^Rt^ (B). The tetra-acylated major lipid A ion of the parental strain (*m/z* 1,404) is acylated by LpxJ, which catalyzes the addition of C_14_ (*m/z* 1,614) or C_16_ (*m/z* 1,642). The minor ion peak at *m/z* 1,642 shown in panel A is likely the result of minimal constitutive PagP activity in this strain. A complete list of peaks and their raw measurements are reported in Table S1 in the supplemental material.

In E. coli lipid A synthesis, the final fatty acid addition by LpxM requires the prior activity of LpxL, the 2′ secondary acyltransferase (KDO_2_-lauroyl-lipid IV_A_ substrate) (34). In contrast to LpxM's rigid substrate selection, LpxJ has flexibility in its activity. LpxJ of H. pylori can act independently of LpxL activity, while homologs from C. jejuni and W. succinogenes act exclusively on tetra-acylated substrates. LpxJ^Rt^ is shown above to act upon penta-acylated lipid A molecules (Fig. 2), but it is unclear whether this is the only lipid A precursor that is a substrate. To determine the requirement of LpxL activity on the activity of LpxJ, we expressed LpxJ in E. coli strain MKV15b (35), which produces mostly tetra-acylated lipid A lacking secondary acylation on the 2′ and 3′ fatty acids (Fig. 4). We found that LpxJ^Rt^ acts upon tetra-acylated substrate (*m/z* 1,404), transferring C_14_ or C_16_ fatty acids (*m/z* 1,615 and 1,642, respectively). These data suggest that Rickettsia lipid A synthesis does not share the strict operational order of acyl chain incorporation found in E. coli.

### Global analysis reveals conserved LpxJ residues critical for acyl transfer.

**FIG 5:**
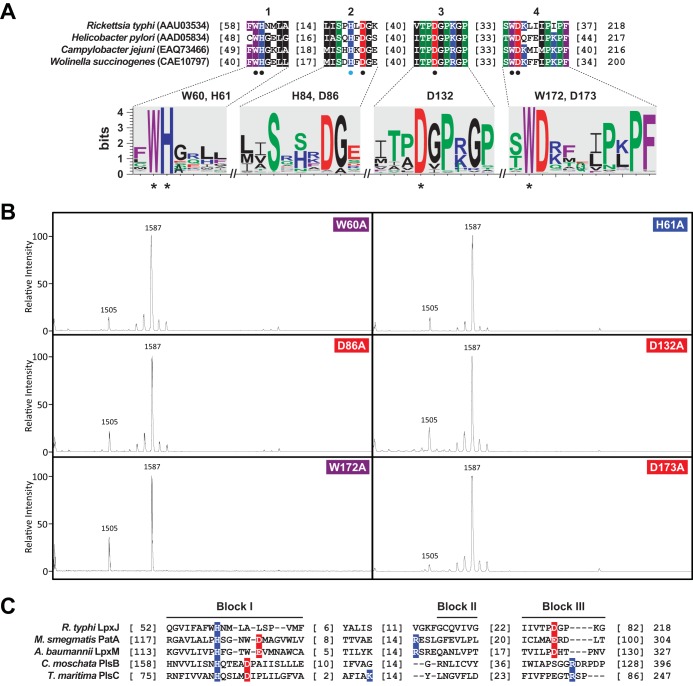
Structural and mutational analysis of LpxJ homologs. (A) Multiple sequence alignment of Rickettsia typhi LpxJ and three Epsilonproteobacteria LpxJ homologs that were previously characterized as lipid A late acyltransferases (31). NCBI protein accession numbers are in parentheses. Only the four most conserved regions of the alignment are shown (numbered 1 to 4 above the sequences). Amino acid coloring is as follows: black, hydrophobic; red, negatively charged; green, hydrophilic; purple, aromatic; blue, positively charged. Black and light blue circles below indicate critical and noncritical residues, respectively, as determined by mutagenesis, shown in panel B. Below each conserved region is a sequence logo (57) depicting conservation across 2,842 compiled LpxJ homologs. Asterisks denote the four residues invariant across all LpxJ homologs. (B) The histidine, aspartic acid, or tryptophan residue at the indicated position in the primary sequence of LpxJ was mutated to alanine, and each construct was individually expressed in MLK1067. Loss of these highly conserved residues abolished the acyltransferase activity of the enzyme, reverting the lipid A phenotype to that of the background strain (penta-acyl, *m/z* 1,587). Mutation of histidine at position 84 to alanine or serine had no effect on enzymatic activity (data not shown). (C) Comparison of R. typhi LpxJ to four divergent lipid acyltransferases. Proteins with associated structures were obtained from the Protein Data Bank: Mycobacterium smegmatis PatA (PDB code 5F34), Acinetobacter baumannii LpxM (5KNK), Cucurbita moschata PlsB (1IUQ), and Thermotoga maritima PlsC (5KYM). R. typhi LpxJ was modeled to all four acyltransferase structures using Phyre2 (58) and fitted to an existing structural alignment template (37), which follows the convention established for naming conserved blocks within GPAT, LPAAT, DHAPAT, and LPEAT acyltransferases (59, 60). Active-site residues for each structure are colored. For LpxJ, Asp132 is proposed to participate in the active site charge relay system with His61 (36, 61).

Analysis of 2,842 distinct LpxJ homologs identified four conserved regions (Fig. 5A). Interestingly, none of these regions contained an HX_4_D/E motif, the hallmark acid/base catalytic mechanism, or charge relay system, that defines glycerol-3-phosphate acyltransferase (GPAT), lysophosphatidic acid acyltransferase (LPAAT), dihydroxyacetone-phosphate acyltransferase (DHAPAT), and 2-acyl-glycerophosphoethanolamine acyltransferase (LPEAT) (36). To gain insight on residues possibly comprising an alternative charge relay system in the LpxJ^Rt^ active site, we mutated conserved His [H61A and H84(A/S)] and Asp (D86A, D132A, and D173A) residues within these regions, as well as two highly conserved Trp residues (W60A and W172A). Aside from H84(A/S), these mutants lacked the enzymatic function of LpxJ^Rt^ when expressed in MLK1067, reverting the lipid A phenotype to that of the background strain (penta-acyl, *m/z* 1,587) (Fig. 5B). The negligible effect on LpxJ^Rt^ activity observed by mutating H84 to either Ala or Ser (data not shown) indicates that H61 is the likely catalytic base of LpxJ.

Provided that all three Asp mutants abolished LpxJ activity, we compared LpxJ to four divergent lipid acyltransferases for which structures have been solved (Fig. 5C). Modeling LpxJ^Rt^ (RT0047) to these structures consistently positioned H61 within the canonical charge relay system (block I). Fitting LpxJ^Rt^ to a structural alignment template (37) positioned D132 with other negatively charged residues in PatA (block III). This suggests that LpxJ is similar to lipid acyltransferases, which have diverged from the canonical HX_4_(D/E) motif by completing the charge relay system across blocks I and III (Fig. 5C). Unlike PatA, however, LpxJ does not have a second positively charged residue (block II) coordinating in the active site, which likely explains the lack of a conserved Asp or Glu within block I for LpxJ. This indicates further divergence of its charge relay system and distinguishes LpxJ from any known lipid acyltransferase.

## DISCUSSION

**FIG 6:**
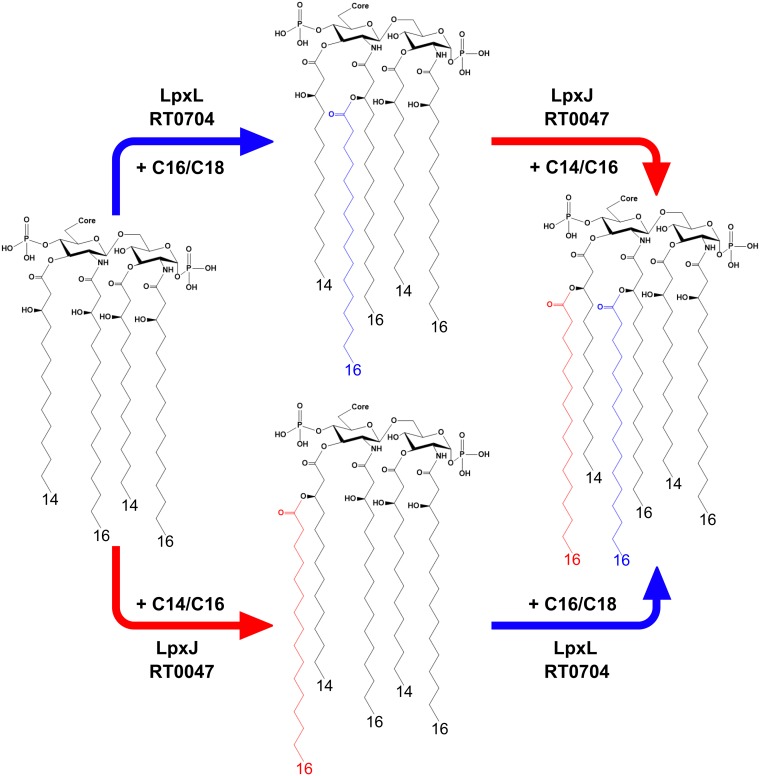
Late acyltransferase activity for R. typhi. The bidirectional additions of the final two fatty acids to complete the biosynthesis of lipid A at the conclusion of the Raetz pathway are shown. Blue arrows indicate LpxJ-mediated acylation, and red arrows indicate LpxL-mediated acylation.

Rickettsia species are obligate intracellular bacterial parasites that produce a typical Gram-negative envelope with the IM and OM separated by a thin peptidoglycan layer (4, 5). Considering the invasive lifestyle of these parasites, the OM sits at the nexus of the host-pathogen interface and is rich in LPS, the classical Gram-negative PAMP (24). Understanding the interplay between mammalian and bacterial molecules at the host-pathogen interface is critically important for the development of novel therapeutic approaches. Considering the historical role of LPS as a mediator of inflammation and its location at the front line of host-pathogen interactions, it is safe to assume that LPS plays a role during Rickettsia infection. However, despite the apparent importance of LPS, there exists a paucity of information about the biology of LPS biosynthesis and its contribution to virulence. In order to address this gap in our understanding of this molecule, we have begun foundational work elucidating the basics of LPS biogenesis in Rickettsia. Here, we characterize new members of the recently discovered LpxJ family of lipid A acyltransferases from R. typhi and R. rickettsii. Using an E. coli reporter system, we have identified LpxJ^Rt^ and LpxJ^Rr^ to be lipid A acyltransferases in Rickettsia. These enzymes catalyze the transfer of secondary fatty acids, predominately C_14_ or C_16_, to the 3-hydroxyl group of the 3′ primary acyl chain. Interestingly, LpxJ^Rt^ shows no preference for either penta- or tetra-acylated lipid A in our reporter system. This implies that activity of the enzyme is bispecific for either lipid IV_A_ or acyl-lipid IV_A_ and can act before LpxL (RT0704), similar to the previously characterized LpxJ homolog from H. pylori. These data indicate that multiple paths exist for secondary acylation of Rickettsia lipid A (Fig. 6) and that secondary fatty acid diversity can be more extensive than described by Fodorová et al. (30). Our analysis of R. typhi lipid A shown in Fig. S4 in the supplemental material indicates a minor lipid A peak at *m/z* 1,907 that is characteristic of shorter fatty acid chain incorporation than that of the major peak at *m/z* 1,936 depicted in Fig. 1. It is conceivable that the C_14_/C_16_ promiscuity observed for LpxJ activity can be contributing to this *in vivo* heterogeneity. Further, both LpxJ homologs characterized here catalyze the addition of fatty acids with odd chain lengths, represented by the peak at *m/z* 1,810 in Fig. 2A and B. This addition is likely an artifact as neither E. coli nor Rickettsia spp. naturally produce odd chain fatty acids (4, 38). It is possible that the presence of short-chain fatty acid metabolites in the growth medium causes nonnatural fatty acid synthesis leading up to cell lysis, as E. coli does produce some odd-chain-length fatty acids when grown in the presence of propionic acid (39). It is also possible that overexpression of rickettsial LpxJ in a heterologous E. coli system is influencing fatty acid biosynthesis and causing erroneous incorporation of odd-length, short-chain fatty acids as building blocks instead of the typical condensation of malonyl-coenzyme A (CoA) during fatty acid manufacturing.

The requirement for Kdosylation of lipid IV_A_ prior to LpxJ^Rt^ activity was not tested here. The E. coli pathway requires KDO sugars be present on lipid IV_A_ prior to secondary acylation by LpxL and LpxM (40). However, previously characterized LpxJ homologs did not share this requirement (31). The combination of fatty acid selection (C_14_-C_16_) and substrate promiscuity (tetra/penta-acyl lipid IV_A_) is unique in comparison to the three previously characterized LpxJ homologs, further expanding the diversity of LpxJ family acyltransferase activity.

Our comparative genomics analysis indicates that LpxJ homologs (all members of DUF374) are widespread across Bacteria (Fig. 5A). These enzymes have functions analogous to the function of the canonical LpxM acyltransferase, and their discovery fills a long-standing hole in the Raetz pathway of lipid A biosynthesis in LpxM-deficient bacteria. Indeed, *in silico* structural prediction of LpxJ^Rt^ indicates superficial similarities across LpxJ and PatA, a recently crystallized member of the LPLAT superfamily that contains an active site deviating from sites of canonical lipid acyltransferases (Fig. 5C). Further, we identified six highly conserved residues in all LpxJ homologs, which we confirmed were necessary for LpxJ function (Fig. 5A and B). These include a histidine at position 61 and an aspartate at position 132 that we believe represent an active-site catalytic dyad similar to PatA. Although these amino acids are distant in the primary sequence, structural modeling predicts a close spatial arrangement of these two residues (37, 41) (Fig. 5C). It is likely that these residues are positioned sufficiently close to allow charge interactions, abstracting a proton from the acceptor hydroxyl and facilitating nucleophilic attack of the incoming acyl chain thioester bond. This putative charge relay system is supported by the recently published crystal structures of PatA from Mycobacterium smegmatis (37, 41), which does not contain the classical HX_4_D motif that is found in other lipid A acyltransferases (42–44) but is absent in LpxJ. Based on the conservation of the proposed catalytic residues within DUF374-containing acyltransferases, this active-site orientation might be common within the LpxJ family.

Lipid A architecture is fundamental to bacterial OM integrity. In many Gram-negative pathogens, changes in lipid A structure can have a profound impact on virulence (45, 46). Therefore, we infer that Rickettsia LpxJ is also important in maintaining ideal membrane dynamics and facilitating molecular interactions at the host-pathogen interface that are required for adhesion and invasion of mammalian cells. Additionally, lipid A is the endotoxic component of LPS (47), and activation of TLR4 is critical for bacterial clearance in mouse models of Rickettsia infection (27, 48), as well as other intracellular parasites (49–51). These data, as well as a recent report highlighting host-specific differences in lipid A gene expression (52), suggest a role for LpxJ in Rickettsia virulence, making this enzyme a tempting target for mutational studies *in vivo*. Further work promises to reveal novel insights into Rickettsia pathogenesis and contribute greatly to our understanding of rickettsial OM physiology.

## MATERIALS AND METHODS

### Recombinant DNA techniques, bacterial strains, and growth conditions.

Primers used in this study were obtained from Integrated DNA Technologies (IDT) and are listed in Table 2. Genomic DNA was isolated from R. typhi grown in Vero76 tissue monolayer as previously described (53). *lpxJ^Rt^* (RT0047; NCBI accession number AAU03534) was amplified using Q5 polymerase 2× master mix (M0492; NEB) and gel purified (740609.50; Macherey-Nagel) before infusion cloning (639649; Clontech) into a *lac*-inducible pFLAG-ctc vector (discontinued; Sigma) using HindIII and XhoI restriction sites. Expression of rickettsial genes after induction was verified by immunoblot detection of the C-terminal FLAG epitope tag. E. coli cultures in mid-log growth phase were induced with 0.5 mM isopropyl-β-d-thiogalactopyranoside (IPTG) for 90 min at 42°C with shaking (225 rpm). Induced cultures and uninduced control cultures were harvested by centrifugation at 4,895 × *g* for 15 min and suspended in 1× NuPAGE Bolt sample buffer containing 1× NuPAGE reducing agent at a concentration of 100 μl of sample buffer for every 1 ml of culture. Samples were heated to 70°C for 10 min and sonicated briefly to fragment genomic DNA. Proteins were separated by SDS-PAGE and transferred to polyvinylidene difluoride (PVDF) membranes, where recombinant LpxJ proteins were detected by indirect chemiluminescent immunoblot analysis using anti-FLAG (F1804; Sigma) primary antibody at a 1:5,000 dilution, followed by goat anti-mouse horseradish peroxidase (HRP)-conjugated secondary antibody (405306; BioLegend) (see Fig. S3 in the supplemental material). The resulting constructs are designated pFLAG-*lpxJ^Rt^* and pFLAG-*lpxJ^Rr^*. Site-directed mutagenesis reactions of selected residues within pFLAG-*lpxJ^Rt^* were carried out using a QuikChange Lightning kit (210519; Agilent). Primers for mutagenesis are listed in Table 2, and protein expression was verified as described above.

**TABLE 2:** E. coli strains used in this study

Strain	Genotype or description	Reference or source
MKV15b	*Δlpx*M *Δlpx*L *Δlpx*P	35
MLK1067 (CGSC 7701)^*a*^	λ^−^ *lpxM11*(Ω)::Cm IN(*rrnD-rrnE*)*1 rph-1*	34
MLK+LpxJ	MLK1067 transformed with pFLAG vector carrying RT0047 (LpxJ^Rt^) or A1G_00705 (LpxJ^Rr^)	This work
MLK+JH61A	MLK+LpxJ^Rt^ with His61 mutated to alanine	This work
MLK+JH84A	MLK+LpxJ^Rt^ with His84 mutated to alanine	This work
MLK+JW60A	MLK+LpxJ^Rt^ with Trp60 mutated to alanine	This work
MLK+JD86A	MLK+LpxJ^Rt^ with Asp86 mutated to alanine	This work
MLK+JD132A	MLK+LpxJ^Rt^ with Asp132 mutated to alanine	This work
MLK+JW172A	MLK+LpxJ^Rt^ with Trp172 mutated to alanine	This work
MLK+JD173A	MLK+LpxJ^Rt^ with Asp173 mutated to alanine	This work

a CGSC, Cole Genetic Stock Center, Yale University.

Bacterial strains used in this study are listed in Table 3. From frozen stocks, E. coli was diluted 1:100 after overnight growth into LB broth supplemented with 1 mM magnesium and containing the appropriate antibiotics. Diluted cultures were then grown with shaking (225 rpm) at 37°C to mid-log phase. Protein expression was induced by addition of IPTG to a final concentration of 1 mM. Induction was carried out at 42°C with shaking (60 rpm) for 90 min. Whole cultures were harvested by centrifugation at 5,000 rpm for 10 min, the supernatants were discarded, and the pellets were either snap-frozen in liquid nitrogen before lipid A microextraction or lyophilized prior to fatty acid analysis.

**TABLE 3:** Primers used in this study

Primer application and name^*a*^	Primer sequence (5′→3′)^*b*^
Cloning	
LpxJ_to_pFLAG.Fw	ATATCATATGAAGCTTATGCGAAAAGCTCTTAAAAAATTTTTAAAAAATAGTAAATGCT
LpxJ_to_pFLAG.Rv	CCCGGGAATTCTCGA**CC**TTTCTTTAAGCTCTCTGTTAAGCTTTTTAATTGT
SDM	
SiteMut_H61toA_top	GGTGTAATCTTTGCATTTTGG**GC**TAATATGCTTGCCTTAAGTCCC
SiteMut_H61toA_bottom	GGGACTTAAGGCAAGCATATTA**GC**CCAAAATGCAAAGATTACACC
SiteMut_H84toA_top	ATCTATGCTTTAATATCACCA**GC**TTTAGATGGTAAAATTTTAAAC
SiteMut_H84toA_bottom	GTTTAAAATTTTACCATCTAAA**GC**TGGTGATATTAAAGCATAGAT
SiteMut_D86toA_top	TATCTATGCTTTAATATCACCACATTTAG**C**TGGTAAAATTTTAAACGCCATAGTAGGGA
SiteMut_D86toA_bottom	TCCCTACTATGGCGTTTAAAATTTTACCA**G**CTAAATGTGGTGATATTAAAGCATAGATA
SiteMut_D132toA_top	CAAGGTGCAAATATAATAGTTACACCGG**C**TGGTCCTAAAGGACCTGTATATAAAGTAAA
SiteMut_D132toA_bottom	TTTACTTTATATACAGGTCCTTTAGGACCA**G**CCGGTGTAACTATTATATTTGCACCTTG
SiteMut_D173toA_top	CTTCTAGGTATTTCAGATTAAAAAGTTGGG**C**TAAATTAATAATACCAATACCGTTTGGT
SiteMut_D173toA_bottom	ACCAAACGGTATTGGTATTATTAATTTA**G**CCCAACTTTTTAATCTGAAATACCTAGAAG
SiteMut_W60toA_top	AAATGAACAAGGTGTAATCTTTGCATTT**GC**GCATAATATGCTTGCCTTAAGTCCCGTTA
SiteMut_W60toA_bottom	ATAACGGGACTTAAGGCAAGCATATTATGC**GC**AAATGCAAAGATTACACCTTGTTCATTT
SiteMut_W172toA_top	TACTTCTAGGTATTTCAGATTAAAAAGT**GC**GGATAAATTAATAATACCAATACCGTTTG

a SDM, site-directed mutagenesis.

b Boldface letters represent nucleotides changed in site-directed mutagenesis.

### Lipid A microextraction.

Microextraction of lipid A from E. coli cultures was performed as previously described (54, 55). Briefly, pellets from 5 ml of mid-log phase E. coli, grown and induced as described above, were extracted in 400 μl of a solution containing five parts of isobutyric acid and three parts of 1 M ammonium hydroxide and heated at 100°C for 1 h, followed by a 15-min incubation on ice and centrifugation at 2,000 × *g* for 15 min. Supernatant was collected and mixed in equal parts with water and then frozen and lyophilized. Contaminants were washed from the dried material by two rounds of methanol washes using 1 ml of methanol, followed by sonication and pelleting at 10,000 × *g* for 5 min. The final product was reconstituted in 2:1:0.25 chloroform-methanol-water (50 μl) along with 4 to 8 grains of Dowex ion exchange resin (Fisher Scientific, Pittsburgh, PA) and incubated with vortexing for at least 5 min. Solubilized lipid A molecules (1 to 2 μl) were spotted onto a stainless steel target plate along with 1 μl of Norharmane matrix (10 mg/ml in 2:1 chloroform-methanol) for MALDI analysis on a Bruker MicroFlex matrix-assisted laser desorption ionization–time of flight (MALDI-TOF) mass spectrometry instrument in negative-ion mode calibrated with Agilent tuning mix (G2421A; Santa Clara, CA), and data were processed using flexAnalysis software (Bruker Daltonics). All microextraction chemicals were obtained from Sigma-Aldrich unless otherwise noted.

### GC fatty acid analysis.

LPS fatty acids were converted to fatty acid methyl esters (FAMEs) and analyzed using gas chromatography flame ionization detection (GC-FID) as previously described (56). Briefly, lyophilized bacterial cell pellets from 50-ml cultures prepared as described above were incubated at 70°C for 1 h in 500 μl of 90% phenol and 500 μl of water. Samples were then cooled on ice for 5 min and centrifuged at 9,391 × *g* for 10 min. The aqueous layer was collected, and 500 μl of water was added to the lower (organic) layer and incubated again. This process was repeated two additional times, and all aqueous layers were pooled. Two milliliters of diethyl ether (E-138-1; Fisher) was added to the harvested aqueous layers. This mixture was then vortexed and centrifuged at 2,095 × *g* for 5 min. The upper (organic) phase was then aspirated off, and 2 ml of diethyl ether was added back to the remaining aqueous phase. The mixture was vortexed and centrifuged at 2,095 × *g* for 5 min, and the lower (aqueous) phase was collected and then frozen and lyophilized overnight. LPS fatty acids were converted to fatty methyl esters in the presence of 10 μg of pentadecanoic acid (P-6125; Sigma) as an internal standard, using 200 μl of 2 M methanolic HCl (Alltech, Lexington, KY) at 90°C for 18 h. Samples were cooled to room temperature, and 200 μl of NaCl-saturated water was added. Converted fatty methyl esters were then extracted twice with hexane and run on an HP 5890 series 2 gas chromatograph. Retention times were correlated to fatty acids using GC-BAME standards (1114; Matreya, Pleasant Gap, PA).

### *In silico* analysis.

To evaluate Rickettsia typhi RT0047 (NCBI locus tag AAU03534) as an LpxJ homolog, we aligned it with previously characterized LpxJ proteins: Helicobacter pylori (AAD05834), Campylobacter jejuni (EAQ73466), and Wolinella succinogenes (CAE10797). To further assess conserved regions of LpxJ proteins, we retrieved 2,842 putative homologs from the NCBI nonredundant protein database in blastp searches using RT0047 as the query. Proteins were aligned, with four conserved regions further evaluated for conservation using WebLogo (57). Both above-mentioned multiple sequence alignments were constructed using MUSCLE (31) (default parameters). Finally, we compared RT0047 to four divergent lipid acyltransferases that have associated structures: Mycobacterium smegmatis PatA (PDB accession number 5F34), Acinetobacter baumannii LpxM (PDB accession number 5KNK), Cucurbita moschata PlsB (PDB accession number 1IUQ), and Thermotoga maritima PlsC (PDB accession number 5KYM). R. typhi LpxJ was modeled to all four acyltransferase structures using Phyre2 (58) and fitted to an existing structural alignment template (37), which follows the convention established for naming conserved blocks within GPAT, LPAAT, DHAPAT, and LPEAT acyltransferases (59, 60).

## Supplementary Material

Supplemental file 1
