# Supplementary material for: Rickettsia Lipid A Biosynthesis Utilizes the Late Acyltransferase LpxJ for Secondary Fatty Acid Addition
Source: J Bacteriol. 2018 Sep 10;200(19):e00334-18. doi: 10.1128/JB.00334-18 (PMC6148475; doi:10.1128/JB.00334-18)
Supplement: Supplemental file 1 [file zjb999094873s1.pdf]

SUPPLEMENTARY MATERIAL

FIG. S1

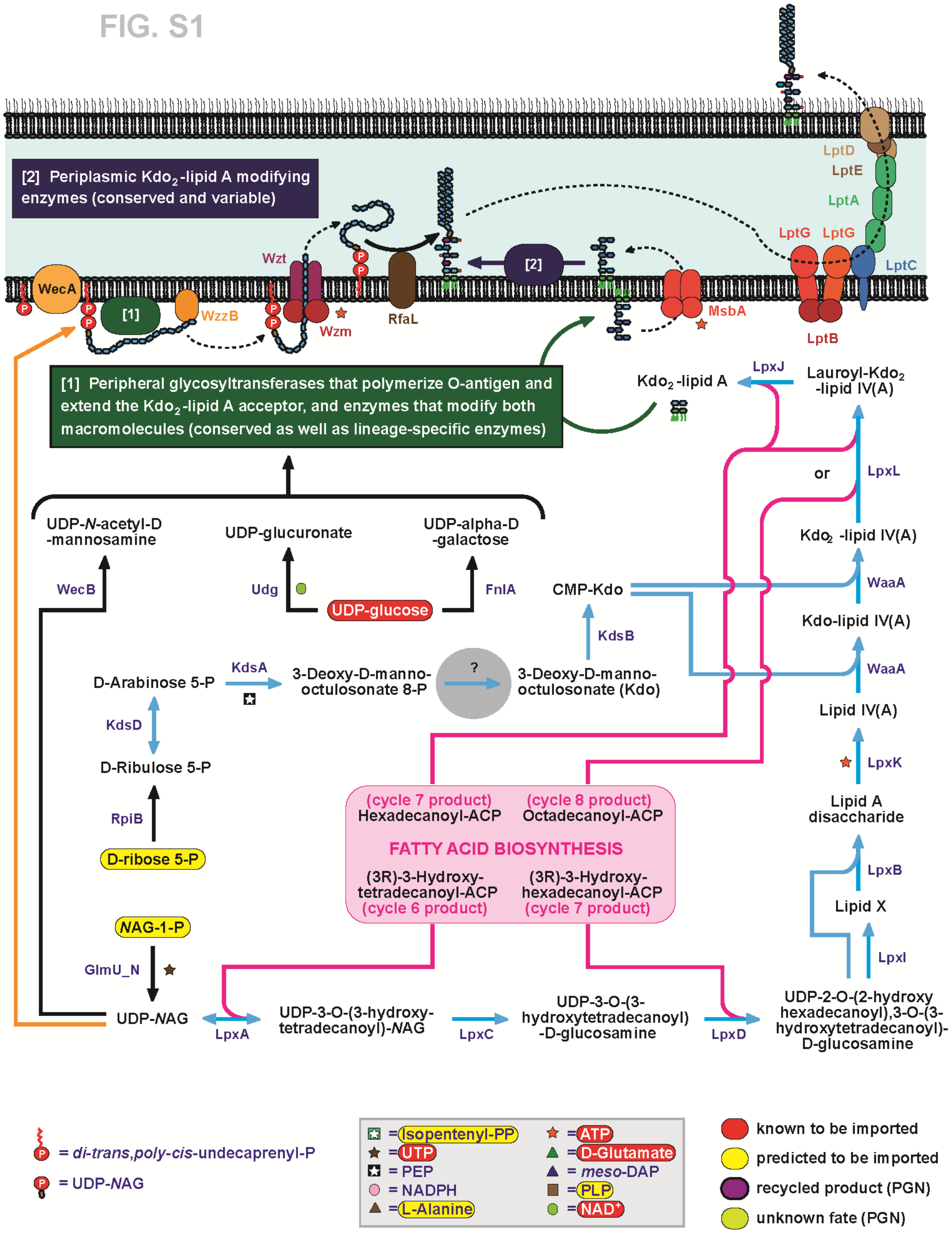

FIG S1. Lipopolysaccharide biosynthesis by *Rickettsia* spp. requires import of *N*AG-1-P, D-ribose 5-P and UDP-glucose from the host. GlmU_N converts imported *N*AG-1-P to UDP-*N*AG, which enters the Raetz pathway (1) for synthesis of Kdo_2-lipid A (light blue arrows). Imported D-ribose 5-P is converted to D-ribulose 5-P via ribose-5-phosphate isomerase B (RpiB), leading to synthesis of CMP-Kdo. A pathway hole (gray circle) indicates the absence of a phosphatase to convert 3-deoxy-D-manno-octulosonate 8-P to 3-deoxy-D-manno-octulosonate (Kdo) (2). The incorporation of various fatty acids into the growing lipid A moiety is shown (pink arrows). Acyl chain incorporation into Lipid A follows the structure deduced for *R*. *typhi* (3). UDP-*N*AG is also ligated to *di-trans*,*poly-cis*-undecaprenyl-P via undecaprenyl-P alpha-*N*-acetylglucosaminyl 1-P transferase (WecA) (orange arrows), creating the lipid carrier for O-antigen. UDP-*N*-acetylglucosamine 2-epimerase (WecB) also utilizes UDP-*N*AG to generate UDP-*N*-acetyl-D-mannosamine (UDP-ManNAc). Imported UDP-glucose is converted to UDP-glucuronate (UDP-GlcA) and UDP-alpha-D-galactose (UDP-Gal) via UDP-glucose 6-dehydrogenase (Udg) and the epimerase/dehydratase FnlA, respectively. UDP-ManNAc, UDP-GlcA and UDP-Gal are predicted to be the main sugars used by peripheral enzymes (e.g., glycosyltransferases, glucosyltransferases, etc.) that polymerize O-antigen and extend the Kdo_2-lipid A acceptor.

Fig S2

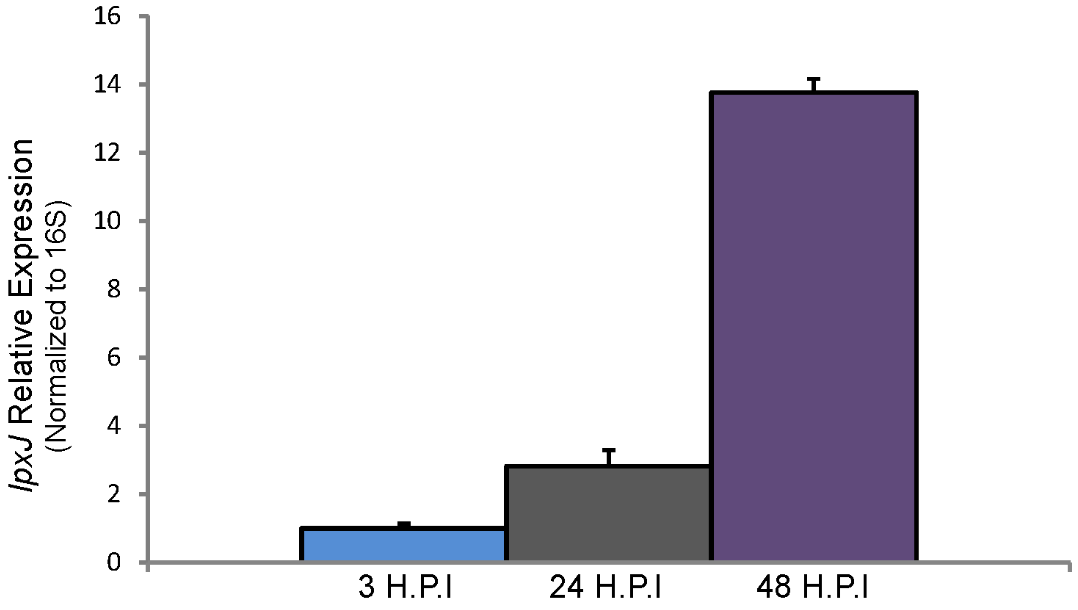

FIG S2. *lpxJ* (Rt0047) is expressed in *R. typhi*. Vero76 cultures infected with *R. typhi* (MOI ~100:1) were incubated at 34°C for 3, 24, and 48 hours post-infection (H.P.I.). RNA was extracted and the relative level of *lpxJ* expression was determined by RT-qPCR using the ΔΔCq method. Data were normalized to expression of the *16s rRNA* gene and plotted +/- SEM. 3 H.P.I. time point was set to = 1 and used as a calibrator for the 24 H.P.I and 48 H.P.I samples.

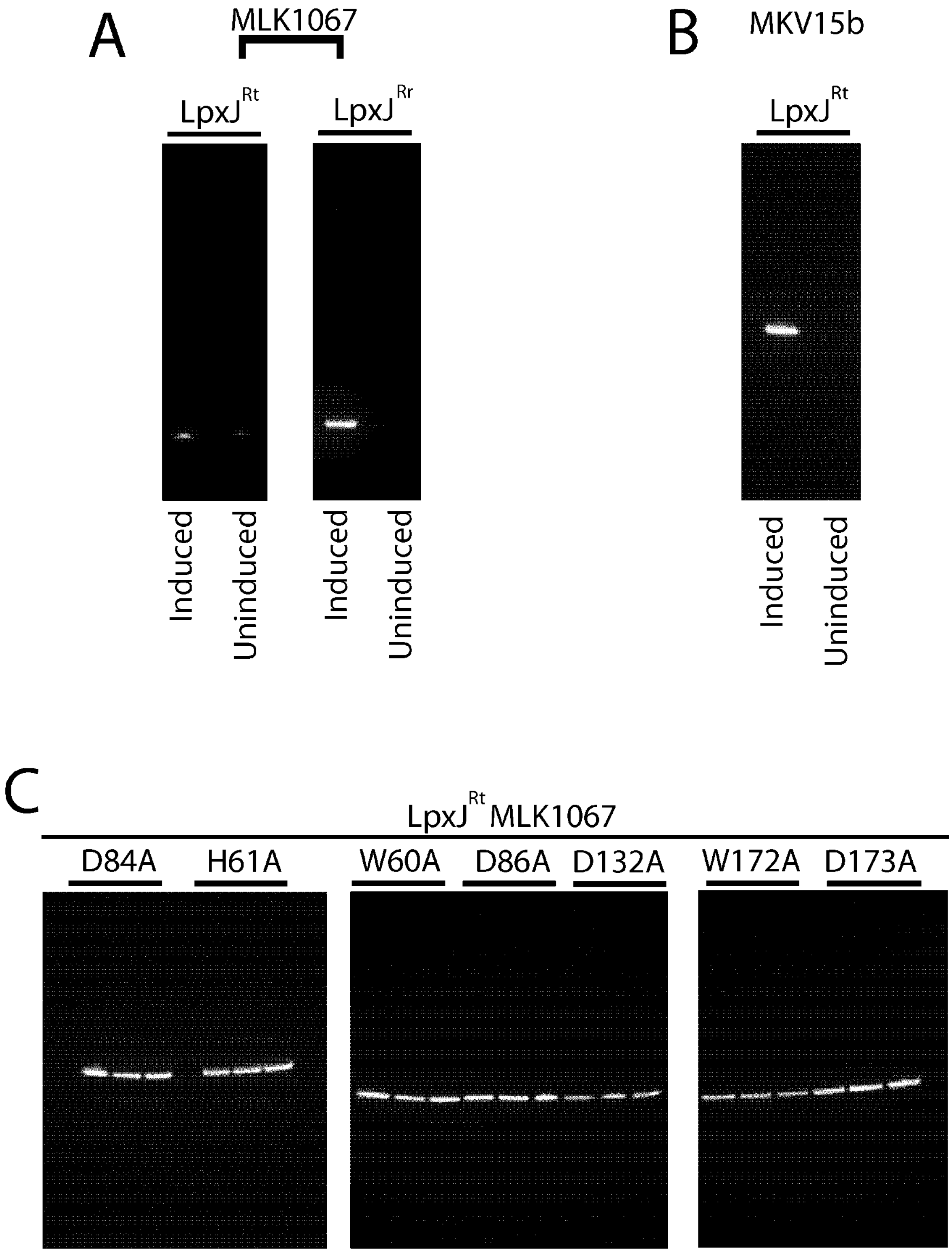
A
MLK1067
LpxJ^{Rt}
LpxJ^{Rr}
Induced
Uninduced
Induced
Uninduced
B
MKV15b
LpxJ^{Rt}
Induced
Uninduced
C
LpxJ^{Rt} MLK1067
D84A
H61A
W60A
D86A
D132A
W172A
D173A

FIG S3. LpxJ^{Rt} and LpxJ^{Rr} ectopic expression in *E. coli* mutants. Whole cellular lysates from *E. coli* strain MLK1067 (A) or MKV15b (B) carrying plasmids encoding the indicated LpxJ homologs were separated by SDS-PAGE and analyzed by immunoblot for the expression of LpxJ using anti-FLAG antibody. (C) Immunoblot detection of LpxJ^{Rt} mutants expressed in MLK1067; 3 colonies each for the indicated point mutation.

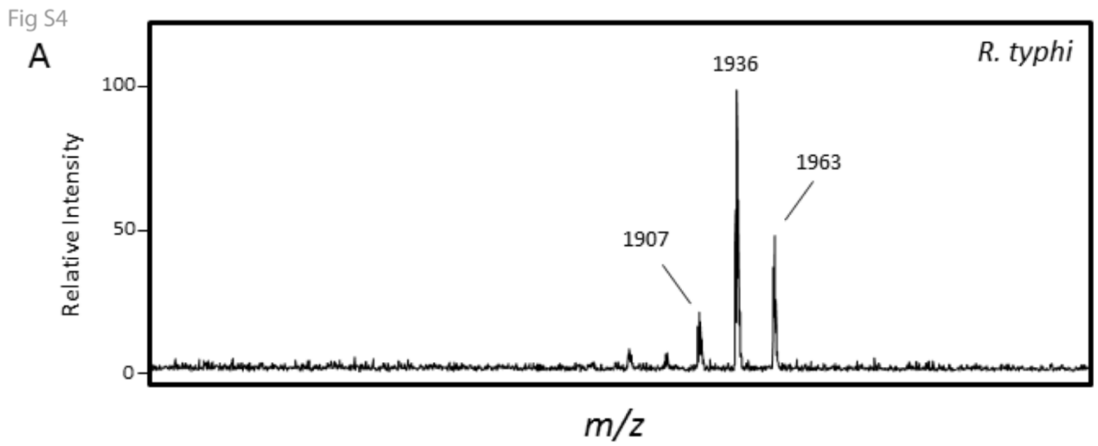

FIG S4. Lipid A spectrum for *R. typhi* str. Wilmington. MALDI analysis of lipid A of *R. typhi* str. Wilmington grown in Vero76 cells. The major peak at m/z 1936 represents the major lipid A structure predicted in reference (3) and depicted in Figure 1. The minor peak at m/z 1963 represent a C16 to C18 switch in fatty acid incorporation at the 2'-secondary acyl chain also described in (3). The minor peak at m/z 1907 has not been described previously, but could represent additional heterogeneity of fatty acid incorporation possibly due to our observed C14/C16 promiscuity of LpxJ.

Table S1. Mass spectrometry data for major and minor peaks observed in Figure 4

Pannel A - MKV15b and empty vector

m/z	S/N	Quality Fac.	Res.	Intens.	Area
**1324.11**	16	4034	2689	225	278
**1376.146**	10	3438	2747	147	205
**1404.131**	139	26748	2084	2103	3797
**1642.352**	30	5885	2565	481	978

Pannel B - MKV15b expressing LpxJRt

m/z	S/N	Quality Fac.	Res.	Intens.	Area
**1322.18**	7	4147	2813	96.3	121
**1348.172**	28	10139	2427	428	683
**1350.335**	14	2119	2483	215	285
**1362.238**	10	5456	2614	159	233
**1374.325**	14	1005	2685	217	287
**1376.301**	49	103839	1888	745	1493
**1388.309**	7	626	2641	105	134
**1390.301**	8	2231	2022	121	196
**1402.321**	22	364	2717	336	427
**1404.188**	22	1019	1271	331	871
**1534.419**	16	12193	2289	245	503
**1562.495**	17	16651	2115	263	594
**1600.465**	8	10481	2219	127	284
**1614.532**	141	79722	1612	1990	6208
**1628.532**	24	34316	1873	363	982
**1642.581**	134	129327	1617	1932	6128
**1853.199**	21	1635	1203	374	1793
**1881.219**	18	2043	1318	319	1422

1. **Whitfield C**, **Trent MS**. 2014. Biosynthesis and export of bacterial lipopolysaccharides. Annu. Rev. Biochem. **83**:99–128.

2. **Driscoll TP**, **Verhoeve VI**, **Guillotte ML**, **Lehman SS**, **Rennoll SA**, **Beier-Sexton M**, **Rahman MS**, **Azad AF**, **Gillespie JJ**. 2017. Wholly *Rickettsia* ! Reconstructed Metabolic Profile of the Quintessential Bacterial Parasite of Eukaryotic Cells. MBio **8**:e00859-17.

3. **Fodorová M**, **Vadovič P**, **Toman R**. 2011. Structural features of lipid A of Rickettsia typhi. Acta Virol. **55**:31–44.
